# Adrenal Myelolipoma: 369 Cases From a High-Volume Center

**DOI:** 10.3389/fcvm.2021.663346

**Published:** 2021-09-10

**Authors:** Lede Lin, Lina Gong, Liang Cheng, Zhihong Liu, Sikui Shen, Yuchun Zhu, Liang Zhou

**Affiliations:** Department of Urology, West China Hospital, Sichuan University, Chengdu, China

**Keywords:** adrenal myelolipoma, hypertension, surgery, remission, connections

## Abstract

**Background:** Adrenal myelolipoma (AML) is a nonfunctional benign neoplasm from the adrenal cortex, composed of mature fat and hematopoietic tissue. Usually, patients have no symptoms. However, some patients with hypertension and blood pressure normalize after AML surgery, indicating some connections between AML and hypertension.

**Materials and Methods:** This was a retrospective cohort study of 369 patients diagnosed with AML from September 2008 to December 2018 collected in the Urology Department of West China Hospital, Chengdu, Sichuan, China. We collected clinical records of patients before surgery. Postoperative follow-up was also carried out for those with hypertension and whether patients needed to take antihypertensive drugs and postoperative blood pressure were recorded. We aim to explore the characteristics of both patients with AML having hypertension and having remission of hypertension in 1 year after surgery.

**Results:** There were 369 patients with AML included in the study, 156 men and 213 women, aged 49.86 ± 11.61 years old. Among them, 121 (32.8%) patients presented with hypertension. Body mass index was significantly higher in the hypertension group than that in the nonhypertension group, even after adjusting other variables (26.26 ± 3.43 vs. 24.28 ± 3.38 kg/m^2^, *P* < 0.001 for both univariate and multivariate analyses). Sixty patients were followed up for 1–9 years, with a median follow-up of 52 months. The duration of hypertension in the remission group was shorter than that in the non-remission group (*P* = 0.020), and the tumor lateralization was significantly different between the two groups (*P* = 0.005).

**Conclusions:** Nearly one-third of patients with AML suffered from hypertension in our study, and there existed some potential links between AML and hypertension. To be more specific, AML-related hypertension was more likely to result from obesity and renal compression by perirenal fat than from endocrine disorders or blood vessels compression. Patients with AML and with more than 3 years of hypertension might have less possibility to recover.

## Introduction

Adrenal myelolipoma (AML) is generally considered a non-functional benign tumor originated from the adrenal cortex, made up of a variable proportion of mature fat and hematopoietic tissues (1). The number of case reports about AML has considerably increased in recent years, which may be related to the development and wide application of imaging techniques, resulting in an increase in the detection of adrenal incidentaloma including AML (2). Female patients are more common, and the predilection age is 50–70 years old, with right lesions more prevalent than those in the left (1, 3). Initially, the neoplasm is asymptomatic, which is discovered during routine image examination. However, some patients presented with lumbar pain, abdominal pain, dizziness, abnormal level of hormones, and so on may be eventually diagnosed as AML under pathological examination after the operation (4, 5). Some patients are present with hypertension and are eventually diagnosed as AML by histopathological biopsy. There have been several reports on the normalized blood pressure following surgery of AML resection, suggesting that there may be a relationship between hypertension and AML (5–8). A previous study reported that the incidence of hypertension accounted for about 22.3% among patients with AML (5). Hence, we aim to explore the characteristics of both patients with AML having hypertension and having remission of hypertension in 1 year after surgery.

## Materials and Methods

### Participants

This was a retrospective cohort study of 369 patients diagnosed with AML from September 2008 to December 2018 collected in the Urology Department of West China Hospital, Chengdu, Sichuan, China. The study was approved by the Ethics Committee of West China Hospital of Sichuan University (IRB approval number 2019708). Informed consent was exempted because all clinical records were acquired retrospectively from the hospital inpatient system (HIS) when patients were admitted to the hospital for diagnosis and treatment, without revealing the privacy of patients. All patients were treated with surgery.

### Including and Excluding Criteria

**Figure 1:**
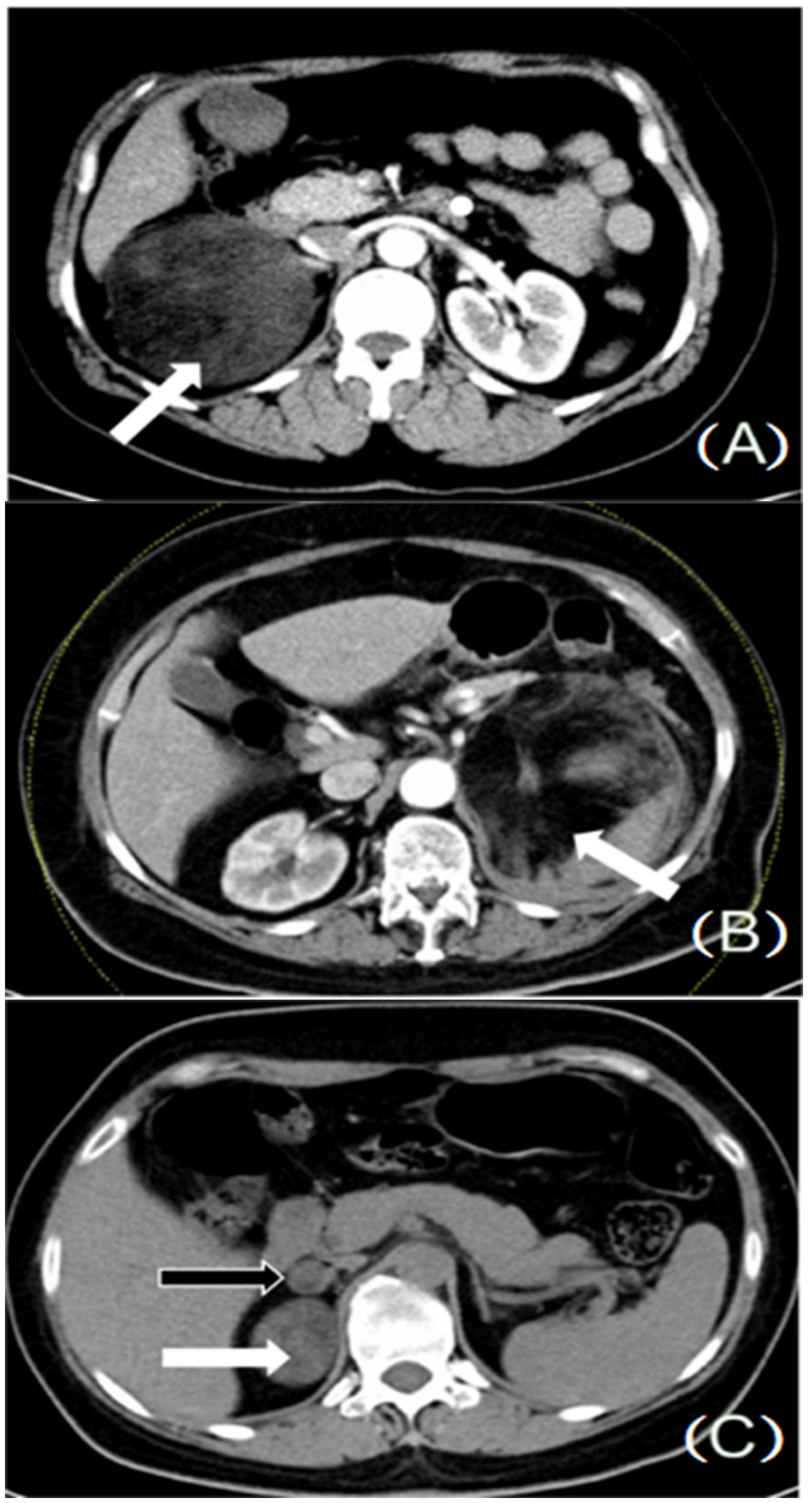
**(A)** Huge adrenal neoplasm, located on the right side (white arrow). **(B)** Huge adrenal neoplasm, located on the left side (white arrow). **(C)** Multiple adrenal neoplasms, located on the right side (white and black arrows).

**Figure 2:**
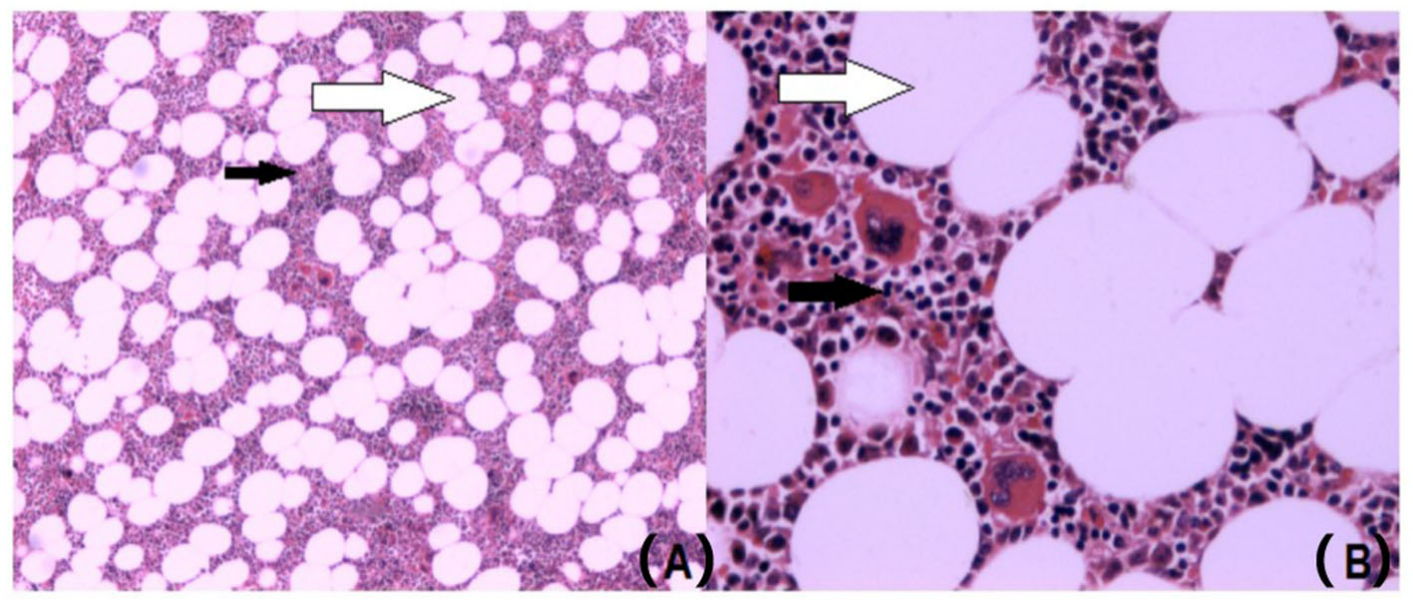
AML is made up of mature fat tissue (white arrow) and hematopoietic tissues (black arrow) in the pathological histological specimen. **(A)** Hematoxylin-eosin staining of AML (×50). **(B)** Hematoxylin-eosin staining of AML (×200), AML, adrenal myelolipoma.

The including criteria of this study were as follows: (1) neoplasm from adrenal presented by computer tomography (Figure 1); (2) adrenal incidentaloma more than 4 cm or complicated with hypertension or other symptoms; (3) patients who underwent surgical removal of adrenal neoplasm; and (4) diagnosis of AML confirmed by pathological examination after surgery (Figure 2).

The excluding criteria of this study were as follows: (1) diagnosis of other tumors by postoperative pathological examination, such as pheochromocytoma, primary aldosteronism (PA), and other adrenal diseases that may cause hypertension; (2) diagnosis of congenital adrenal hyperplasia and primary adrenocortical hypofunction.

### Data

We collected clinical records of patients before surgery, consisting of gender, age, leading complaints (namely, lumbar pain and abdominal pain), history of diabetes, tumor lateralization, tumor diameter, body mass index (BMI), duration of hypertension, blood pressure, the number of antihypertensive drugs, preoperative plasma renin activity (PRA), and plasma aldosterone concentration (PAC). Postoperative follow-up was also carried out for those with hypertension and whether patients needed to take antihypertensive drugs and postoperative blood pressure were recorded.

### Group

All eligible patients were divided into two groups: hypertension and non-hypertension, based on the complication of hypertension (systolic blood pressure ≥140 mmHg or diastolic blood pressure ≥90 mmHg measured three times on 2 different days, or having started antihypertensive therapy). Based on the outcomes of follow-up, those with hypertension were divided into two groups: the remission group and the non-remission group. The remission of hypertension after the operation is defined as stopping the use of antihypertensive drugs for 3 months and the blood pressure coming down to normal (<140/90 mmHg), which lasts till the end of follow-up.

### Statistical Analysis

All the procedures were finished on the statistical software, IBM SPSS Statistics 22. Discrete variables were reported as a percentage and continuous variables as mean ± SD or median (quartile). For analysis of categorical variables, Pearson's chi-square test or Fisher's exact test was used. Differences between means were tested with Student's *t*-test. The multivariate logistic regression model was utilized to test selective variables in previous univariate analyses. The receiver operation characteristic (ROC) curve was utilized to examine the efficacy of a diagnostic model. *P* < 0.05 was applied to certify the statistical significance between the two groups of data. The normality of the distribution was tested using the Shapiro–Wilk test.

## Results

### Characteristics of Patients With AML

There were 369 eligible patients with AML enrolled in our study, comprising 156 men and 213 women. Around 10 patients were with open surgery. The average age was 49.86 ± 11.61 years, among which 121 patients were complicated with hypertension. The duration of hypertension ranged from 1 day to 30 years, and the mean systolic and diastolic pressures in the hypertension group are 168.89 ± 23.18 and 99.29 ± 14.82 mmHg, respectively. Antihypertensive drugs included nifedipine, amlodipine, captopril, irbesartan, metoprolol, prazosin, furosemide, and so on. The incidence of hypertension in AML is 32.8%.

### Characteristics of AML With Hypertension

Data were analyzed in the subgroup of age, and the demographic and clinical features were detailed in Table 1; Supplementary Table 1. We found that the percentage of hypertension in each subgroup increased with age. BMI was significantly higher in the hypertension group than that in the non-hypertension group, even after adjusting other variables (26.26 ± 3.43 vs. 24.28 ± 3.38 kg/m^2^, *P* < 0.001 for both univariate and multivariate analyses). In addition, BMI was significantly higher in almost all subgroups, except for subgroups 20–29 and 60–79 (Supplementary Table 1). The lateralization in the hypertension group was significantly different from that in the non-hypertension group for univariate analysis (Table 1). However, there was no significant difference in the tumor lateralization in multivariate analysis and in each subgroup (Table 1; Supplementary Table 1). Unfortunately, we were only able to retrieve preoperative PRA and PAC data of 272 patients. We discovered that the hypertension group had more preoperative endocrine disorders, namely, suspicious PA [defined as PAC >15 ng/dl and PAC/PRA > 25 (ng/dl)/(ng/ml·h)] and suspicious secondary aldosteronism [SA, defined as PAC > 15 ng/dl and PRA > 4.5 ng/(ml·h)] (Table 1).

**Table 1:** Characteristics of patients with AML having hypertension.

**Demographic and clinical features**	**Hypertension**	**Non-hypertension**	**Univariate *P* value**	**Multivariate *P* value**
Number of cases (%)	121 (32.8%)	248 (67.2%)		
Mean age ± SD, years	54.88 ± 10.13	47.42 ± 11.52	<0.001	<0.001
Sex, males/females	54/67	102/146	0.523	
Mean BMI ± SD, kg/m^2^	26.26 ± 3.43	24.28 ± 3.38	<0.001	<0.001
Leading complaints (%)	27 (22.3%)	46 (18.5%)	0.394	
Diabetes mellitus (%)	16 (13.2%)	19 (7.6%)	0.087	0.898
Tumor location, left/right/bilateral	54/67/0	73/168/7	0.004	0.782
Mean tumor diameter ± SD, cm	6.11 ± 3.02	5.74 ± 2.85	0.244	
Mean PRA, ng/(ml·h)	2.60 ± 3.18	2.71 ± 2.68	0.771	
Mean PAC, ng/dl	16.78 ± 6.31	15.92 ± 6.33	0.271	
Suspicious PA^*^	20/101	13/171	<0.001	
Suspicious SA^*^	18/101	20/171		
PA and SA negative^*^	63/101	138/171		

*AML, adrenal myelolipoma; BMI, body mass index; PA, primary aldosteronism; PAC, plasma aldosterone concentration; PRA, plasma renin activity; SA, secondary aldosteronism; SD, standard deviation. Leading complaints include lumbar pain and abdominal pain*.

* *We were only able to retrieve preoperative PRA and PAC data in 272 patients. Suspicious PA was defined as PAC > 15 ng/dl and PAC/PRA > 25 (ng/dl)/(ng/ml·h). Suspicious SA was defined as PAC > 15 ng/dl and PRA > 4.5 ng/(ml·h)*.

### Characteristics of Hypertension Remission in 1 Year After Surgery

Among 121 cases of AML complicated with hypertension, we finished following up 60 patients with a follow-up time of 1–9 years. The median follow-up time was 52 months. There were 16 patients not using drugs in 1 year after the operation, whose blood pressure returned to normal and continued till the end of the first year. One patient stopped taking antihypertensive drugs after the operation; nonetheless, the blood pressure rose again in 3 weeks, and blood pressure was controlled well by taking antihypertensive drugs, while 43 patients needed to continue to take antihypertensive drugs after the operation, and the blood pressure was well controlled. The number of antihypertensive drugs and level of blood pressure are not significantly different before and after surgery.

There was a significant difference in the lateralization of tumor between the remission group and the non-remission group, with a *P* value < 0.05 (Table 2). As for the duration of hypertension, we utilized the Wilcoxon rank sum test to ensure the inspection efficacy, proving that the statistical difference was significant. The remission rate was 26.67% in 1 year after the surgical treatment, while 22.22% (10 out of 45 cases) in 3 years, and 7 cases of 28 cases were with normal blood pressure value in the 5-year follow-up after operation. Cases with normal blood pressure had no further potential increase in blood pressure requiring antihypertensive medication.

**Table 2:** Characteristics of hypertension in 1 year after surgery.

**Demographic and clinical features**	**Remission**	**Non-remission**	***P* value**
Number of cases (%)	14^#^(23.3%)	46 (76.7%)	
Mean age ± SD, years	54.14 ± 10.01	54.89 ± 10.09	0.809
Sex, males/females	7/7	17/29	0.383
Mean BMI ± SD, kg/m^2^	24.95 ± 2.32	25.86 ± 3.63	0.273
Leading complaints (%)	2 (14.3%)	8 (17.4%)	1.000
Median duration of hypertension (quartile), y	0.75 (0.08, 2)	4.5 (0.42, 8.25)	0.015
Tumor location, left/right	1/13	23/23	0.004
Mean tumor diameter ± SD, cm	5.37 ± 2.50	6.03± 2.79	0.436
Mean PRA, ng/(ml·h)	1.48 ± 1.50	2.50 ± 3.15	0.153
Mean PAC, ng/dl	16.01 ± 3.75	16.35 ± 6.07	0.820
Suspicious PA^*^	3/10	8/38	0.328
Suspicious SA^*^	0/10	7/38	
PA and SA negative^*^	7/10	23/38	

*BMI, body mass index; PA, primary aldosteronism; PAC, plasma aldosterone concentration; PRA, plasma renin activity; SA, secondary aldosteronism; SD, standard deviation. Leading complaints include lumbar pain and abdominal pain*.

# *There were 16 cases in the remission group, however, the blood pressure in two of them rose again in 1 and 2 years after the operation, respectively. Therefore, we put those two cases in the nonremission group*.

* *We were only able to retrieve preoperative PRA and PAC data in 48 patients. Suspicious PA was defined as PAC > 15 ng/dl and PAC/PRA > 25 (ng/dl)/(ng/ml·h). Suspicious SA was defined as PAC > 15 ng/dl and PRA > 4.5 ng/(ml·h)*.

**Figure 3:**
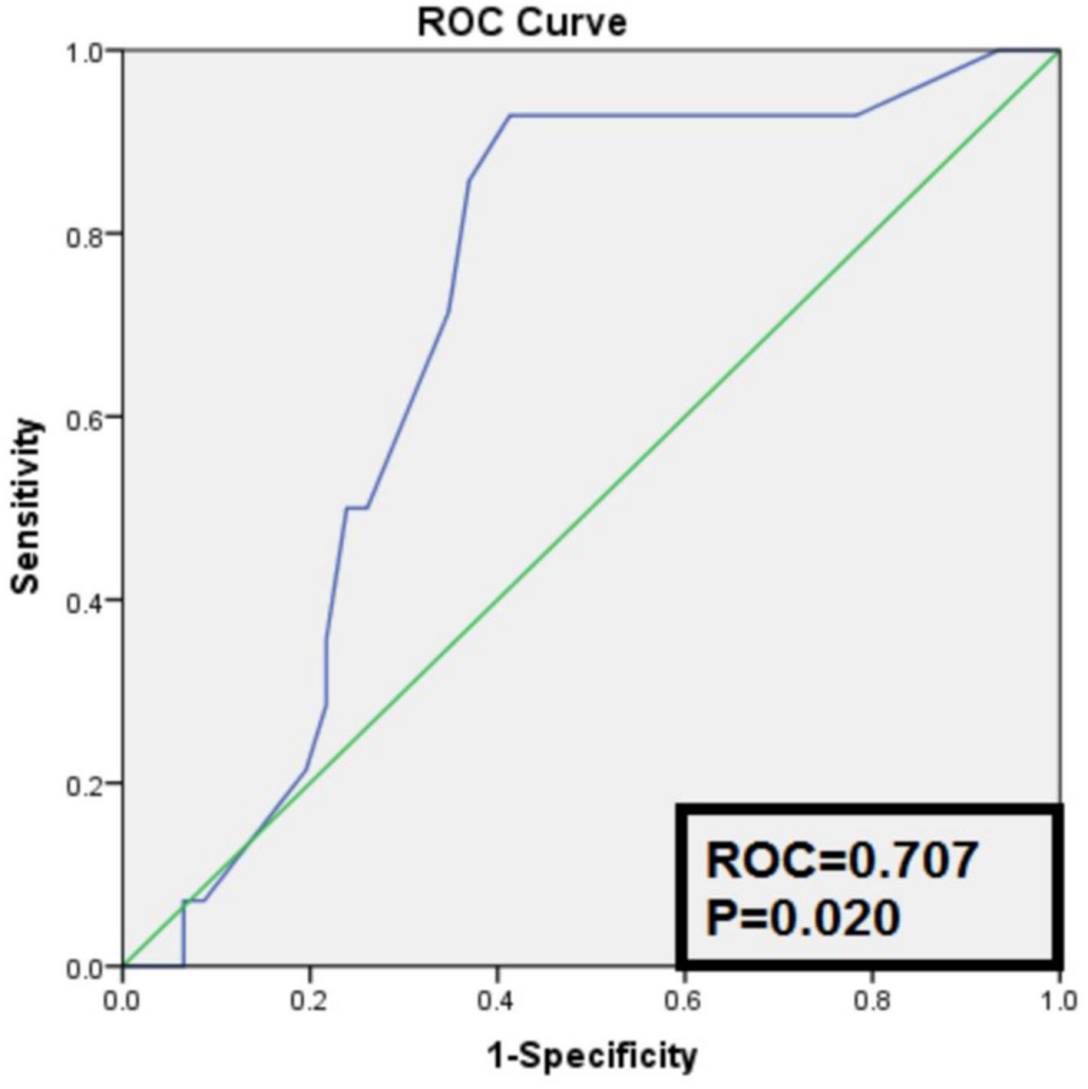
Receiver operation characteristic curve of the duration of hypertension to distinguish remission group from non-remission group.

The ROC curve (Figure 3) was used to test the efficacy of the duration of hypertension to distinguish the remission group from the non-remission group in Table 2, and we were able to see the area under the curve (AUC) is significant, with a *P* value = 0.020. Thirteen out of 14 cases in the remission group were of hypertension duration no more than 3 years and the other one with 10 years of hypertension. The information in Supplementary Table 2 showed that the cutoff of the duration of hypertension as ≤ 3 years distinguished the hypertension remission group from the non-remission group, with a sensitivity of 92.9% and a specificity of 58.7%. The positive predictive value was 40.6%, and the negative predictive value was 96.4%.

We also retrieved preoperative PRA and PAC data of 48 patients among the 60 follow-up patients. We discovered that preoperative suspicious PA accounted for 3/10 and 8/38 patients in the remission group and the non-remission group, respectively. As for patients with preoperative suspicious SA, they were all in the non-remission group (Table 2).

We further analyzed the 48 patients with available hormone data in Table 3. In the suspicious PA subgroup, we were able to find that BMI and the duration of hypertension were both significant between the remission group and the non-remission group (22.39 ± 0.98 vs. 27.62 ± 3.29 for BMI, *P* = 0.028; and 0.5 vs. 5.5 years for the duration of hypertension, *P* = 0.012; Table 3). While in the suspicious SA subgroup, all seven patients in the non-remission group, four of them realized their hypertension duration for more than 1 year, and five of them were considered to be overweight or obese, with a mean tumor diameter of 7.31 ± 5.50 cm. In PA and SA negative groups, although a significant difference was not found, we found that the duration of hypertension and tumor diameter were shorter and smaller in the remission group than those in the non-remission group (1 vs. 3 years for the duration of hypertension and 4.79 ± 1.35 vs. 5.76 ± 2.05 cm for tumor diameter, Table 3).

**Table 3:** Characteristics of hypertension in 1 year after surgery according to preoperative PRA and PAC.

	**Remission**	**Non-remission**	***P* value**
Suspicious PA^*^			
Number of cases (%)	3 (27.3%)	8 (72.7%)	
Mean age ± SD, years	57.00 ± 5.57	54.38 ± 11.08	0.711
Sex, males/females	0/3	2/6	1.000
Mean BMI ± SD, kg/m^2^	22.39 ± 0.98	27.62 ± 3.29	0.028
Leading complaints (%)	0 (0%)	3 (37.5%)	0.491
Median duration of hypertension, y	0.5	5.5	0.012
Tumor location, left/right	0/3	5/3	0.182
Mean tumor diameter ± SD, cm	5.4 ± 2.76	6.13± 2.20	0.658
PA and SA negative^*^			
Number of cases (%)	7 (23.3%)	23 (76.7%)	
Mean age ± SD, years	57.00 ± 10.54	55.57 ± 10.38	0.752
Sex, males/females	5/2	10/13	0.388
Mean BMI ± SD, kg/m^2^	25.54 ± 2.09	26.17 ± 3.92	0.686
Leading complaints (%)	1 (14.3%)	4 (17.4%)	1.000
Median duration of hypertension, y	1	3	0.564
Tumor location, left/right	1/6	13/10	0.126
Mean tumor diameter ± SD, cm	4.79 ± 1.35	5.76 ± 2.05	0.249

*BMI, body mass index; PA, primary aldosteronism; PAC, plasma aldosterone concentration; PRA, plasma renin activity; SA, secondary aldosteronism; SD, standard deviation*.

* *Suspicious PA was defined as PAC > 15 ng/dl and PAC/PRA > 25 (ng/dl)/(ng/ml·h). Suspicious SA was defined as PAC > 15 ng/dl and PRA > 4.5 ng/(ml·h)*.

## Discussion

Adrenal myelolipoma is a rare tumor with fat density under the CT scan and is clinically relevant. Because of the advancement of imaging technology and its popularization, reports of AML have been increased during the last decades (5, 9–14). There exists a dilemma that diagnosis of AML is difficult because of its benign properties and reliability of the pathological examination. In this study, we aim to explore characteristics of both patients with AML having hypertension and having remission of hypertension in 1 year after surgery. Based on our study, patients with AML were usually diagnosed at the age of 49.86 ± 11.61 on average, which is consistent with the report of Decmann A (5), who reviewed 440 cases of AML in literature with a mean age of 51 ± 14.4. The incidence of AML with hypertension in our study is 32.8%, while 22.3% of AML with hypertension was reported by Decmann A (5). It seems that there may exist some intrinsic connections between AML and hypertension.

On the one hand, patients with AML may be complicated with primary hypertension. In this study, we found that the proportion of hypertension in each subgroup in Supplementary Table 1 increased with age, which is also a clinical risk characteristic of primary hypertension (15). If patients with AML are complicated with primary hypertension, implying their blood pressure is not directly related to AML, the possibility of hypertension relief after the operation will be low. After stratified by age, we found that patients in each subgroup had nearly equal age between the hypertension group and the non-hypertension group, with even a slight decrease in age in the hypertension group in subgroups 30–39 and 50–79 (Supplementary Table 1). The phenomenon indicated that age might not be a risk factor in AML-related hypertension.

On the other hand, we discovered that patients in the hypertension group had higher BMI than those in the non-hypertension group (Table 1). When subgroup analysis was made by age, we were also able to observe that all the subgroups had higher BMI in the hypertension group, suggesting BMI tended to be a possible risk factor in AML-related hypertension. Recently, more and more evidence has appeared and illustrated the connections between obesity and primary hypertension (16–21). Excessive weight gain, especially an increase in perirenal fat (PRF) is strongly associated with hypertension (21). As AML is composed of fat tissue, it is considered to serve as a part of PRF and has a potential association with BMI. Excessive BMI might lead to an increased risk of renal compression by PRF. As a result, patients with AML having hypertension had higher BMI and more possibility of renal compression.

Several reports had described that AML might accompany endocrine function and result in hypertension, namely, hyperaldosteronism (22, 23), hypercortisolism (24), hypercatecholaminemia (6–8), and androgen secretion (5). The symptoms typically vanished after surgical removal of AML. In our study, we evaluated preoperative PRA and PAC levels in 272 patients, finding that more suspicious PA and SA were detected in the hypertension group (19.8 vs. 7.6% for the suspicious PA and 17.8 vs. 11.7% for the suspicious SA). However, the postoperative pathological examination revealed AML and only three out of 11 patients with the suspicious PA got remission from the surgery in our follow-up. The phenomenon indicated that only a small proportion of AML contributed to endocrine disorders and not all of them benefited from operation.

We found an interesting phenomenon in our research that more AML were located on the right side, with a proportion of 63.7%. Several studies (1, 25, 26) also reported this phenomenon. The cause of this phenomenon may be attributed to the pathogenesis of AML. Here comes a theory that adipose tissue originating from mesenchymal stem cells harbors in the adrenal cortex and causes inflammatory reaction under certain stimulation, which is the first step in forming AML (27). In our human body, the right adrenal gland faces more possibility of friction from the inferior border of the liver when we breathe, which serves as a sort of stimuli contributing to the appearance of AML. However, the pathogenesis is even more complicated and perhaps involves hormonal pathways. Further experimental data may reveal the reason why there is a dominant right-sided AML.

In our study, we found that the important characteristic of hypertension remission in 1 year after surgery was the duration of hypertension. The shorter the duration of hypertension, the greater the possibility of blood pressure returning to normal after the operation. After the lesion is surgically removed, the compression effect on the kidney is relieved, and hypertension is alleviated. However, the blood pressure of some patients did not return to normal, and we think, apart from primary hypertension, AML may compress the renal artery, leading to inflammation and promoting fibrosis, which causes irreversible damage to kidneys and blood vessels. If so, the preoperative PRA and PAC will elevate. But we only observed seven out of 48 patients had preoperative suspicious SA, all in the non-remission group, suggesting that this is not the main reason why blood pressure did not normalize. To go a step further, we analyzed the 48 hormone-available patients and discovered that, in the suspicious PA subgroup, patients had larger BMI and longer hypertension duration in the non-remission group than in the remission group (*P* = 0.028 for BMI and *P* = 0.012 for the duration of hypertension, Table 3). In addition, in the suspicious SA subgroup, 5/7 patients in the non-remission group were overweight or obese. While for PA and SA negative subgroups, although BMI in the non-remission group was slightly higher, we found that hypertension duration and tumor diameter were longer and larger than those in the remission group. This is following the aforementioned evidence, that is, obesity and PRF have a close association with hypertension (16–21). Chronic obesity and longer PRF compression contribute to resistant hypertension. Thus persistent hypertension may also be associated with the long duration of renal compression, leading to renal ischemia, increased intrarenal pressure, sodium reabsorption, and even lipotoxicity (16). The pathophysiological process may be irreversible when it exists for a prolonged period. Nevertheless, BMI also has a significant disadvantage in that it is not equal to visceral adipose tissue and PRF since the indicator contains muscle components and cannot differentiate them from the adipose tissues (16). So, the BMI difference in the PA and SA negative subgroups was not significant. In Supplementary Table 2, we set a cutoff of ≤ 3 years to predict if AML with hypertension will relieve after the operation. If a patient undergoes AML surgery, whose hypertension duration is more than 3 years, his possibility of remission is relatively low, partially due to primary hypertension or the long duration of renal compression.

Table 2 also presented a thought-provoking outcome. In the remission group, almost all cases (13/14) were from the right side. We discovered that the mean tumor diameter in the remission group was smaller than that in the non-remission group, but not significantly. Was this phenomenon attributed to a lack of samples or some reasons like the anatomical difference between left and right adrenal glands in our human body? We still do not know and so more clinical and experimental data are needed.

This study had several advantages. First, it provided the largest number of AML in the literature up till now. We depended on our central hospital, one of the most famous central hospitals in southwestern China with adrenal surgeries around 300–400 per year, to conduct such research. Second, we discovered that AML-related hypertension was more likely to result from obesity and renal compression by PRF than from endocrine disorders or blood vessels compression. Third, we obtained a duration cut-off that AML-related hypertension with more than 3 years had less possibility to recover, which was beneficial for clinical management of patients with AML having hypertension. Last, we put forward several interesting phenomena about AML lateralization and its links with hypertension remission.

The study also had some shortcomings, unfortunately. This was retrospective-designed research, and we were unable to retrieve hormone data after the operation because most of the patients did not return for follow-up. Instead, they would usually be followed up in local hospitals, which reflected the diagnosis and treatment system in our country. On the other hand, as AML is a rare disease entity, the best way to explore its features is to design such a retrospective study. And we think that this study did add to a more comprehensive understanding of AML and hypertension.

## Conclusions

Nearly one-third of patients with AML suffered from hypertension in our study and there existed some potential links between AML and hypertension. To be more specific, AML-related hypertension was more likely to result from obesity and renal compression by PRF than from endocrine disorders or blood vessels compression. Patients with AML and with more than 3 years of hypertension might have less possibility to recover.

## Data Availability Statement

The original contributions presented in the study are included in the article/Supplementary Material, further inquiries can be directed to the corresponding author/s.

## Ethics Statement

The studies involving human participants were reviewed and approved by the Ethics Committee of West China Hospital of Sichuan University. Written informed consent for participation was not required for this study in accordance with the national legislation and the institutional requirements.

## Author Contributions

LL, LG, and LZ conception and design of the study. LL, LG, LC, ZL, SS, and YZ acquisition of data (laboratory or clinical). LL, LC, and LZ data analysis and/or interpretation. LL, YZ, and LZ drafting of the manuscript and/or critical revision. YZ and LZ approval of the final version of manuscript. All authors contributed to the article and approved the submitted version.

## Funding

The work was supported by the 1.3.5 project for disciplines of excellence, West China Hospital, Sichuan University (ZY2016104), the Innovation Spark Project of Sichuan University (2018SCUH0061), and the National Natural Science Fund of China (81800667).

## Conflict of Interest

The authors declare that the research was conducted in the absence of any commercial or financial relationships that could be construed as a potential conflict of interest.

## Publisher's Note

All claims expressed in this article are solely those of the authors and do not necessarily represent those of their affiliated organizations, or those of the publisher, the editors and the reviewers. Any product that may be evaluated in this article, or claim that may be made by its manufacturer, is not guaranteed or endorsed by the publisher.

## Abbreviations

AML: Adrenal myelolipoma
BMI: Body mass index
SD: Standard deviation
ROC: Receiver operation characteristic
AUC: Area under curve
PRA: Plasma renin activity
PAC: Plasma aldosterone concentration
PA: Primary aldosteronism
SA: Secondary aldosteronism
PRF: Peri-renal fat.
