# Supplementary material for: Adrenal Myelolipoma: 369 Cases From a High-Volume Center
Source: Front Cardiovasc Med. 2021 Sep 10;8:663346. doi: 10.3389/fcvm.2021.663346 (PMC8462508; doi:10.3389/fcvm.2021.663346)
Supplement: Supplementary file 1 [file Table_1.DOCX]

**Supplement Table 1. Characteristics of AML patients with hypertension**

| **Demographic and clinical features** | **Hypertension** | **Non-hypertension** | **P value** |
| --- | --- | --- | --- |
| **Number of cases(%)** | 121(32.8%) | 248(67.2%) |  |
| **Age 20-29, n=21** | 1 | 20 |  |
| **Mean age ± SD, years** | 29 | 26.35 ±2.16 | 0.246 |
| **Sex, males/females** | 1/0 | 11/9 | 1.000 |
| **Mean BMI ± SD, kg/m^2^** | 33.26 | 26.12±4.39 | 0.135 |
| **Leading complaints(%)** | 0 | 6(30.0%) | 1.000 |
| **Diabetes mellitus(%)** | 0 | 0 |  |
| **Tumor location, left/right/bilateral** | 1/0/0 | 3/17/0 | 0.190 |
| **Mean tumor diameter ± SD, cm** | 3 | 5.40±3.24 | 0.479 |
| **Mean PRA, ng/(mL·h)** | 7.8 | 5.28±3.82 | 0.533 |
| **Mean PAC, ng/dL** | 16.86 | 18.16±6.96 | 0.859 |
| **Suspicious PA*** | 0/1 | 0/15 | 0.269 |
| **Suspicious SA*** | 1/1 | 5/15 |  |
| **PA and SA negative*** | 0/1 | 10/15 |  |
| **Age 30-39, n=41** | 4 | 37 |  |
| **Mean age ± SD, years** | 34.50±2.65 | 34.70±3.17 | 0.903 |
| **Sex, males/females** | 4/0 | 15/22 | 0.082 |
| **Mean BMI ± SD, kg/m^2^** | 26.63±0.92 | 24.35±3.47 | 0.023 |
| **Leading complaints(%)** | 1(25.0%) | 4(10.8%) | 0.418 |
| **Diabetes mellitus(%)** | 0 | 2(5.4%) | 1.000 |
| **Tumor location, left/right/bilateral** | 1/3/0 | 6/30/1 | 0.866 |
| **Mean tumor diameter ± SD, cm** | 5.13±2.25 | 5.78±2.81 | 0.653 |
| **Mean PRA, ng/(mL·h)** | 5.79±3.67 | 3.25±3.12 | 0.205 |
| **Mean PAC, ng/dL** | 17.97±4.37 | 16.57±7.51 | 0.721 |
| **Suspicious PA*** | 0/3 | 0/23 | 0.182 |
| **Suspicious SA*** | 2/3 | 5/23 |  |
| **PA and SA negative*** | 1/3 | 18/23 |  |
| **Age 40-49, n=120** | 37 | 83 |  |
| **Mean age ± SD, years** | 45.65±2.73 | 44.60±2.76 | 0.057 |
| **Sex, males/females** | 19/18 | 38/45 | 0.573 |
| **Mean BMI ± SD, kg/m^2^** | 26.18±3.10 | 24.60±3.15 | 0.031 |
| **Leading complaints(%)** | 10(27.0%) | 17(20.5%) | 0.428 |
| **Diabetes mellitus(%)** | 3(8.1%) | 2(2.4%) | 0.343 |
| **Tumor location, left/right/bilateral** | 14/23/0 | 20/60/3 | 0.181 |
| **Mean tumor diameter ± SD, cm** | 6.48±3.43 | 5.54±2.68 | 0.101 |
| **Mean PRA, ng/(mL·h)** | 3.06±3.79 | 2.43±2.34 | 0.418 |
| **Mean PAC, ng/dL** | 17.16±5.03 | 15.31±6.13 | 0.160 |
| **Suspicious PA*** | 6/29 | 2/62 | 0.001 |
| **Suspicious SA*** | 6/29 | 2/62 |  |
| **PA and SA negative*** | 17/29 | 58/62 |  |
| **Age 50-59, n=104** | 33 | 71 |  |
| **Mean age ± SD, years** | 53.79±3.04 | 53.89±2.77 | 0.869 |
| **Sex, males/females** | 14/19 | 27/44 | 0.669 |
| **Mean BMI ± SD, kg/m^2^** | 27.22±3.63 | 23.49±3.12 | ＜0.001 |
| **Leading complaints(%)** | 5(15.2%) | 12(16.9%) | 0.822 |
| **Diabetes mellitus(%)** | 4(12.1%) | 8(11.3%) | 1.000 |
| **Tumor location, left/right/bilateral** | 13/20/0 | 25/43/3 | 0.472 |
| **Mean tumor diameter ± SD, cm** | 5.81±2.77 | 6.07±3.27 | 0.686 |
| **Mean PRA, ng/(mL·h)** | 2.34±3.08 | 2.57±2.44 | 0.711 |
| **Mean PAC, ng/dL** | 16.92±6.76 | 15.59±6.00 | 0.360 |
| **Suspicious PA*** | 7/29 | 5/50 | 0.072 |
| **Suspicious SA*** | 3/29 | 8/50 |  |
| **PA and SA negative*** | 19/29 | 37/50 |  |
| **Age 60-69, n=68** | 35 | 33 |  |
| **Mean age ± SD, years** | 63.06±2.54 | 64.42±3.21 | 0.057 |
| **Sex, males/females** | 12/23 | 11/22 | 0.934 |
| **Mean BMI ± SD, kg/m^2^** | 25.59±3.20 | 23.97±3.47 | 0.105 |
| **Leading complaints(%)** | 9(25.7%) | 6(18.2%) | 0.454 |
| **Diabetes mellitus(%)** | 6(17.1%) | 5(15.2%) | 0.824 |
| **Tumor location, left/right/bilateral** | 19/16/0 | 16/17/0 | 0.632 |
| **Mean tumor diameter ± SD, cm** | 6.31±3.22 | 5.37±2.01 | 0.157 |
| **Mean PRA, ng/(mL·h)** | 2.56±2.75 | 1.50±1.28 | 0.076 |
| **Mean PAC, ng/dL** | 16.87±7.61 | 15.87±5.79 | 0.595 |
| **Suspicious PA*** | 5/29 | 5/20 | 0.032 |
| **Suspicious SA*** | 6/29 | 0/20 |  |
| **PA and SA negative*** | 18/29 | 15/20 |  |
| **Age 70-79, n=15** | 11 | 4 |  |
| **Mean age ± SD, years** | 72.91±2.43 | 73.75±3.59 | 0.608 |
| **Sex, males/females** | 4/7 | 0/4 | 0.454 |
| **Mean BMI ± SD, kg/m^2^** | 24.76±3.74 | 22.79±2.02 | 0.495 |
| **Leading complaints(%)** | 2(18.2%) | 1(25.0%) | 1.000 |
| **Diabetes mellitus(%)** | 3(27.3%) | 2(50.0%) | 0.836 |
| **Tumor location, left/right/bilateral** | 6/5/0 | 3/1/0 | 0.905 |
| **Mean tumor diameter ± SD, cm** | 5.82±1.90 | 8.33±2.15 | 0.047 |
| **Mean PRA, ng/(mL·h)** | 0.73±0.75 | 0.38 | 0.671 |
| **Mean PAC, ng/dL** | 14.56±5.69 | 22.1 | 0.238 |
| **Suspicious PA*** | 2/10 | 1/1 | 0.021 |
| **Suspicious SA*** | 0/10 | 0/1 |  |
| **PA and SA negative*** | 8/10 | 0/1 |  |

AML: adrenal myelolipoma; SD:standard deviation

Leading complaints include lumbar pain and abdominal pain.

*We were only able to retrieve preoperative PRA and PAC data in 272 patients. Suspicious PA was defined as PAC＞15ng/dL and PAC/PRA＞25(ng/dL)/(ng/mL·h). Suspicious SA was defined as PAC＞15ng/dL and PRA＞4.5ng/(mL·h).
