# Supplementary material for: Adrenal Myelolipoma: 369 Cases From a High-Volume Center
Source: Front Cardiovasc Med. 2021 Sep 10;8:663346. doi: 10.3389/fcvm.2021.663346 (PMC8462508; doi:10.3389/fcvm.2021.663346)
Supplement: Supplementary file 2 [file Table_2.DOCX]

**Supplement Table 2. Evaluation of duration of hypertension (≤3 years as a cutoff)**

|  | **Remission** | **Non-remission** | **Total number** |
| --- | --- | --- | --- |
| **≤3 years** | **13** | **19** | **32** |
| **＞3years** | **1** | **27** | **28** |
| **Total number** | **14** | **46** | **60** |
